# Islands and archipelagos: Reconciling programmatic vs. opportunistic research in health professions education

**DOI:** 10.1007/s40037-020-00628-7

**Published:** 2020-11-19

**Authors:** Glenn Regehr

**Affiliations:** Centre for Health Education Scholarship, Faculty of Medicine, University of British Columbia, Vancouver, Canada

In the Insider’s Perspective section, an insider in health professions education offers his/her thoughts, contemplations and advice on readers’ dilemmas or questions. You can send your questions or dilemmas to lieda.meester@bsl.nl. And who knows, your question may be the topic of the next Insider’s Perspective instalment.

Dear insider, I have just finished my PhD and I want to continue in the field of HPE research. Now my professor tells me to develop a clear and recognizable program of research. I need to have a clear career topic, she says. Should I work on a program of research or can I be more opportunistic in my selection of questions to explore?

There are several ways to think about what it means to engage in a program of research. How *you* choose to frame the notion of “programmatic” will affect how you think about the place of opportunistic research in your career. There are indeed some researchers in health professions education who pick a specific topic area and spend the majority of their research time in that area. Some of these individuals focus on a more conceptual issue, such as the nature of expertise in clinical diagnosis and management. Others focus on more concrete problems, such as the development and refinement of a particular educational innovation or assessment tool. Still others find a space that is somewhere in between, such as exploring the nature of entrustment decisions and how to record them (which encompasses both conceptual and practical questions). Yet even these highly focused individuals are likely to stray into other areas of research occasionally, for any number of reasons. Their side forays may be because their job description includes a certain amount of support for others who are hoping to conduct research, even if it is not directly in the researcher’s core topic area. Or it may result from chance opportunities a researcher feels cannot be passed up (one colleague of mine describes occasionally joining research projects because she is excited to work with members of the research team regardless of the particular topic). Because of their deep commitment to some core research area, these focused researchers may draw the distinction between “their” programmatic research (which they actively pursue) and the “other” research activities they engage in opportunistically. Thus, they may try to actively limit the amount of opportunistic research they engage in and keep as much focus as possible on their own research program (an approach that your supervisor seems to be recommending).

My own approach to research has tended to blur the boundaries between programmatic and opportunistic research. There are certain themes that run through my work, but each of those themes arose opportunistically because of my work with others. And each moves forward because a new individual comes to me with an interest in the area and a new perspective on the issue. As an example, “my” self-assessment work began many years ago during a casual walk with a psychiatry resident who, when I asked him what scholarly area he was interested in, said that he wanted to improve students’ self-assessment. The ensuing conversation led us to start to wonder what self-assessment really was, and what it was for. This became the topic for his Master’s thesis, as he developed a new model of self-assessment (something he called the relative ranking model). After a few studies supporting that research program, he moved in different directions. So that area of “my” research lay fallow for a few years until a junior colleague started working in the area and approached me to collaborate with him. This new collaborator led several studies that developed the idea of self-monitoring as a more sensible conceptualization of how clinicians stay safe in practice. During that time, a surgeon colleague was showing interest in understanding the notion of safe practice in surgery, which led to my supporting her PhD thesis exploring self-monitoring in everyday practice (what she called “slowing down when you should”). For me, that area of research has now morphed into explorations of the role of the “self” in feedback because of the interests of other students and collaborators. I could tell similar stories about my research themes around OSCE measures, workplace-based assessment, professional identity construction, or expertise in clinical reasoning. I did not actively pursue any of these areas as “my research”, but when opportunities arose, I was happy to marry the person’s interests with something I had been thinking about. And I only started thinking about that particular area because someone had initially approached me with an interesting problem they wanted to explore. So perhaps the best description of my own research style is that I have a programmatic approach to addressing the practical problems of education that arise opportunistically through my interactions with others.

However, there is also a deeper level of “programmatic” coherence across all my research enterprises: the consistent set of conceptual lenses that I bring to whatever I am working on. I think each of us (or at least most of us) develop our own set of lenses through our disciplinary training. We inherit them, in part, from our mentors and supervisors and, in part, from those we choose to read and follow in the literature. These lenses define the ways in which we tend to frame the world (and its problems). It shapes our ways of interpreting situations, the sorts of questions we naturally ask and, therefore, the sorts of answers we can find. Thus, our conceptual lenses position us relative to others in the field. My own roots are in cognitive psychology, and all the research questions that I develop have this cognitivist framing. So regardless of topic, my focus is always on the individual—how people develop an understanding of the world around them and how that understanding affects their decisions and ways of acting in the world. Others, of course, bring their own lenses: sociology, rhetoric, psychometrics, population sciences, implementation sciences, or critical theory (to name just a few).

These conceptual lenses offer another way of thinking about your “program” of research. The specific problems you address may change fairly regularly (because of the various opportunities that arise) but the lenses you apply to them (the way you frame them and explore them), will be more constant (although these too might evolve with time). By way of analogy, I liken my research approach and my resulting collaborative publication record to islands in an ocean. Each collaborator I work with can legitimately claim ownership of the island we are co-constructing (the particular topic or question). But if one looks at all these islands from 10,000 metres up, it becomes clear that they form an archipelago. My contribution to the work is not just the building of the island, but the conceptual and methodological landmass that sits under the water on which each of the islands are built. If a person’s work is too far away from the archipelago, then it takes a much greater amount of foundational work (pouring material onto the ocean floor) before the island itself (the study) can take shape. But if I can negotiate the person close to my landmass, then it more easily supports the development of the person’s island and it extends my archipelago.

For many of us who come to the field of health professions education from a discipline-based PhD training program, we have never had to explicitly define the shape of that landmass, nor think about how it might effectively support HPE types of islands. It is for this reason that I often recommend to those entering the field that they spend some time writing their own manifesto in order to help them develop a clear understanding for themselves and others what perspective they bring to the field, what their perspective allows them to uniquely see, and how that new insight might help others in the field address the problems they are grappling with. This helps others understand and appreciate the 10,000 metre view of your archipelago (the coherence of your CV, even if it might look like a random collection of topics on the ground). And it helps you make rational choices about what to collaborate on (or how to make a collaboration meaningful if choice is not an option). This manifesto is generally not easy to write and can sometimes take up to a year or two, because it is not just a matter of stating who you are but shaping it in a way that makes sense (and sounds relevant) to an eclectic audience of disciplinarians and practitioners with differing backgrounds, goals and lenses of their own. The point is not to teach others your perspective, but to show them the power of what that perspective can reveal. But if done well, such a document can become the written manifestation of your landmass, and the foundation on which you can co-construct a set of islands programmatically.

So going back to the original question, there is no right answer on how to be successful in HPE research. You can pick a topic area and focus much of your energy on it. Or you can move through the research space more eclectically, finding intriguing problems through your engagement with interesting people. But regardless of which approach you choose, if you wish to contribute well, it does seem sensible that you be reflective about your own ways of thinking about problems and asking questions, and strategically leverage that positionality in a programmatic way.

